# Lamb-Wave-Based Tomographic Imaging Techniques for Hole-Edge Corrosion Monitoring in Plate Structures

**DOI:** 10.3390/ma9110916

**Published:** 2016-11-12

**Authors:** Dengjiang Wang, Weifang Zhang, Xiangyu Wang, Bo Sun

**Affiliations:** School of Reliability and Systems Engineering, Beihang University, No. 37 Xueyuan Road, Haidian District, Beijing 100191, China; wangdengjiang@buaa.edu.cn (D.W.); zhangweifang@buaa.edu.cn (W.Z.); wangxiangyu2016@buaa.edu.cn (X.W.)

**Keywords:** health monitoring, lamb wave, tomographic imaging, ART, corrosion

## Abstract

This study presents a novel monitoring method for hole-edge corrosion damage in plate structures based on Lamb wave tomographic imaging techniques. An experimental procedure with a cross-hole layout using 16 piezoelectric transducers (PZTs) was designed. The A0 mode of the Lamb wave was selected, which is sensitive to thickness-loss damage. The iterative algebraic reconstruction technique (ART) method was used to locate and quantify the corrosion damage at the edge of the hole. Hydrofluoric acid with a concentration of 20% was used to corrode the specimen artificially. To estimate the effectiveness of the proposed method, the real corrosion damage was compared with the predicted corrosion damage based on the tomographic method. The results show that the Lamb-wave-based tomographic method can be used to monitor the hole-edge corrosion damage accurately.

## 1. Introduction

For many important structures in critical infrastructures, such as the wings of planes, load bearing walls, and oil pipelines, the integrity of the structure determines the safety and reliability of the system [1,2]. In order to ensure the integrity of the structure, traditional non-destructive testing (NDT) methods are used, which include X-ray, eddy current, acoustic emission, and others [3,4,5]. Traditional NDT methods are used offline, and the testing devices are expensive and complex. Another disadvantage of traditional NDT is that they often need to destroy the original structures, and periodic NDT will lead to additional expense when the detected structure has no damage. In recent years, structural health monitoring (SHM) was developed based on the application of advanced sensors. SHM techniques can monitor the structure online [6,7,8]. Piezoelectric transducers (PZTs) have been widely used in the field of SHM as they are lightweight, low cost, and leave the layout of the original structures unaffected [9]. For propagating in a thin plate, the Lamb wave is a kind of guide wave, whose wavelength is similar to the thickness of the structure. Lamb waves are widely used in detecting large areas of damage in thin plates [10,11]. Many researchers have focused on the large areas of detection in plate structures [7,12].

Many Lamb-wave-based techniques for localization and quantification of damages in existing structures have been proposed, and the tomography is a typical one [13,14,15,16]. For Lamb-wave-based tomography, many different kinds of sensor configurations have been developed, including parallel, fan beam, and cross-hole configuration [17]. Kevin et al. [15] developed a method based on Lamb waves to localize the defects in the structure. In their research, a square array of PZT sensor networks was posted on an aluminum plate and the algebraic reconstruction technique (ART) was used for imaging the damages. Mahadev et al. [18] used the Lamb waves to detect the damage existing in the composite plate, and they modified the sensor configuration and normalized the energy of Lamb waves for defective specimens with respect to that of the defect-free specimen. Zhao et al. [19] developed a method known as the reconstruction algorithm for probabilistic inspection of defects (RAPID), and they verify the effectiveness of the algorithm based on experiments of the aluminum plates of wings. Zhao et al. [13] also compared several tomographic imaging techniques, which include the filtered back projection (FBP), the ART, and the RAPID. The advantages and drawbacks of these methods, such as reconstruction fidelity, quality, efficiency, and the minimum number of sensors required for each array geometry, are discussed. In reference [20], a damage index (DI) based on the fractal dimension of Lamb waves is proposed using a modified box-counting algorithm, and the proposed DI has been combined with a tomographic imaging algorithm to identify damages in plate-like structures. Huang et al. [21] proposed a new Omni-directional electromagnetic acoustic transducer (EMAT) for the ultrasonic Lamb waves (ULW) tomography imaging (TI) of defects in metallic plates. They also proposed a new cross-hole tomography imaging (CTI) method for variable-depth defects in metal plates based on multi-mode electromagnetic ultrasonic Lamb waves (LWs) [22]. A composite baseline-free delamination inspection technique of composite plates which combine an air-coupled Lamb waves scan method and virtual time reversal (VTR) algorithm is proposed in reference [23].

In practical engineering structures, riveted holes and bosses exist. The area near the hole is the stress-concentrated part, and failure is often caused in this area. Crack damage and corrosion damage are two basic types of damage in structures. For crack damage at the edge of the hole, many researchers studied the localization and quantification of crack damage. Grondel et al. [24] designed an integrated experimental platform based on Lamb waves using piezoelectric transducers. Compared with results obtained using acoustic emission devices, it showed that the platform can identify cracks more effectively. Quaegebeur et al. [25] investigated the correlation-based imaging technique for crack propagation in a riveted aluminum plate. They proposed a method of imaging the damage using the relationship between the measured signals at the elements of the piezoceramic arrays and numerical signals by finite dimension piezoceramics. In reference [26], the damage index was proposed to predict structural cracks. The damage index is defined as the ratio of the scatter energy contained in the S_0_ mode wave package to that of the baseline S_0_ mode wave package. He et al. discovered three signal features of Lamb waves and developed a data-driven model to quantify the crack size at the riveted hole [27]. In reference [28], a novel hybrid sensor, which included PZT sensors and intelligent coating sensors, was proposed to monitor the crack damage at the edge of the hole. The S_0_ mode of the Lamb wave was used, and the normalized amplitude and phase change of S_0_ mode were extracted as damage features.

In practical engineering applications, compared with the crack damage, the hole-edge corrosion damage is also a main failure mechanism for structures, and the corrosion damage at the edge of the hole will accelerate the failure of the structure. The mode of Lamb waves and the extracted signal features of Lamb waves are not the same for different types and situations of damage. However, few research papers deal with monitoring the hole-edge corrosion damage, and an effective technique for monitoring the corrosion damage at the hole is needed. In this paper, the main purpose is to use the tomographic Lamb wave for a new application (the corrosion damage at the edge of the hole) which is meaningful for practical engineering. In this study, the Lamb-wave-based tomographic imaging technique for hole-edge corrosion monitoring is studied. The paper is organized as follows. In Section 2, a brief introduction of PZT sensors is given and the modes of Lamb waves are studied. Then the ART method is developed to locate and image the hole-edge corrosion damage. In Section 3, an experiment is designed to verify the proposed method, and a homogenization method is proposed to process the images. In Section 4, the predicted corrosion damage and the real corrosion damage are compared and discussed. The imaging results show the proposed method can monitor the hole-edge corrosion accurately.

## 2. Methodology Development

### 2.1. Lamb Wave for PZT Sensor

**Figure 1:**
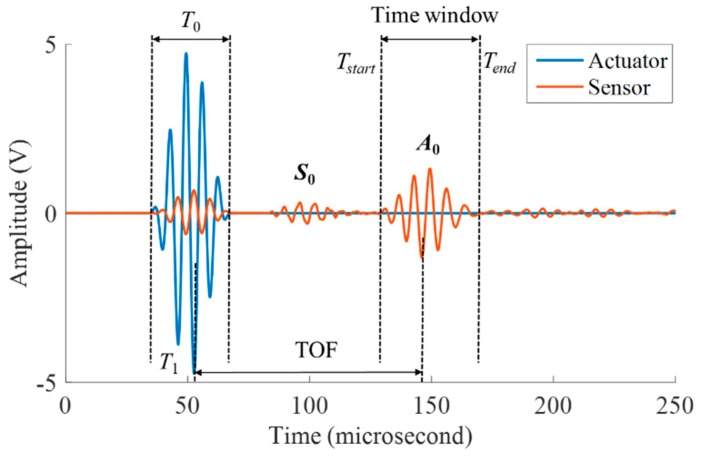
Schematic illustration for the time window calculation.

Lamb-wave-based PZT sensors have shown great potential in non-destructive evaluations and structural health monitoring systems. Lamb waves allow the evaluation of large areas in a short period of time for both isotropic and anisotropic materials [7]. The mechanism of Lamb waves damage quantification is to identify discontinuities in the wave propagation paths that alter the characteristics of transmitted/deflected waves [29]. Compared with the S_0_ mode, the fundamental antisymmetrical mode (A_0_) of Lamb waves can detect thickness-loss damage more accurately [13,30]. In this study, the corrosion damage is a kind of thickness-loss damage, so the first wave package of the A_0_ mode is chosen to extract damage-sensitive features from the data. The group velocity is verified in an undamaged specimen by measuring the time-of-flight (*ToF*) between two sensors with a known distance. The calculated time window is the time duration between *T_start_* and *T_end_*, which are given in Equations (1) and (2), respectively:

(1) $$T_{start}=T_{1}+ToF-\frac{1}{2}T_{0}\;,$$

(2) $$T_{end}=T_{1}+ToF+\frac{1}{2}T_{0}\;,\;\;$$

where *T*_1_ represents the wave excitation time of an actuator, *ToF* is the time of flight from an actuator to a receiver, and *T*_0_ is the period of excitation wave envelope. The time window is illustrated in Figure 1.

For different types of damage, a specific feature should be extracted in order to quantify the damage in the structure. Using measured signal directly for corrosion damage is difficult and data reduction is generally required to extract the damage feature. In the time-domain signal, three signal features are usually used, which include normalized amplitude, phase change, and correlation coefficient [27]. Some other signal features are also constructed based on the specific method [25,31]. In this paper, when corrosion damage exists in the specimen, the thickness of the corrosion area will decrease, and the frequency-thickness of the Lamb waves will decrease accordingly. Based on the group velocity and phase velocity of A_0_ mode, the time window of the received signal will alter to a wider time window and the phase will change. In order to capture the changing Lamb waves based on corrosion damage, the correlation coefficient between the received signal with no damage (namely healthy condition) and the damage were used. Theoretically, the correlation coefficient is a statistical comparison between the signal in the present state and the signal in the reference state. The signal from the actuator-sensor path that has discontinuities is affected by the presence of the damage, and the correlation coefficient between the baseline signal and the signal from the damaged specimen changes. As the damage size increases, the correlation coefficient will be reduced from that of the undamaged specimen [32], and dissimilar intensities of signal changes can be an indicator of the corrosion area. There are also some other practical issues which influence this assumption, including operating temperature, aging of the sensor bonding agent, and sensor coupling variations due to structural vibration. In general, the emphasis of this work is on demonstrating the concept in the laboratory environment where these parameters can be controlled, and some other signal features also can be considered to quantify the hole-edge corrosion damage.

The correlation coefficient, ρ, between two sets of data, $s_{j},\;s_{k}$, is

(3) $$\rho =\frac{\mathrm{Cov}\left(s_{j},s_{k}\right)}{\sigma_{s_{j}}\sigma_{s_{k}}},$$

where the covariance, Cov, is

(4) $$\mathrm{Cov}\left(s_{j},s_{k}\right)=\frac{\sum_{i}^{N}\left(s_{j}\left(t_{i}\right)-{\bar{s}}_{j}\right)\left(s_{k}\left(t_{i}\right)-{\bar{s}}_{k}\right)}{N}$$

and the standard deviations, $\sigma_{s_{j}}$ and $\sigma_{s_{k}}$, are

(5) $$\sigma_{s_{j}}=\sqrt{\sum_{i}^{N}{\left(s_{j}\left(t_{i}\right)-{\bar{s}}_{j}\right)}^{2}}\;\;\;,$$

(6) $$\sigma_{s_{k}}=\sqrt{\overset{N}{\underset{i}{\sum }}{\left(s_{k}\left(t_{i}\right)-{\bar{s}}_{k}\right)}^{2}}\;\;\;\;,$$

A data set in this case refers to waveforms acquired from sensor pairs. The reference data set ($s_{j}$) was acquired immediately after initial installation. The signal data with existing damage were compared to the signal data from a healthy condition to generate tomograms based on the correlation coefficient.

### 2.2. ART Method

In the beginning of the 20th century, Kaczmarz and Cimmino developed iterative algorithms for solving linear systems independently [33]. In 1970, Gordon, Bender, and Herman rediscovered Kaczmarz’s method by applying it in medical imaging [34], and they called the method Algebraic Reconstruction Technique (ART) [35]. When image reconstruction is needed with sparse data, the ART method based on the Kaczmarcz algorithm is a natural choice [33,36]. Many different kinds of sensor configurations have been developed which include parallel, fan beam, and cross-hole [17] configuration. In this study, only a limited number of sensors were attached on the plate, therefore, the conventional cross-hole configuration is optimal and was chosen [13].

**Figure 2:**
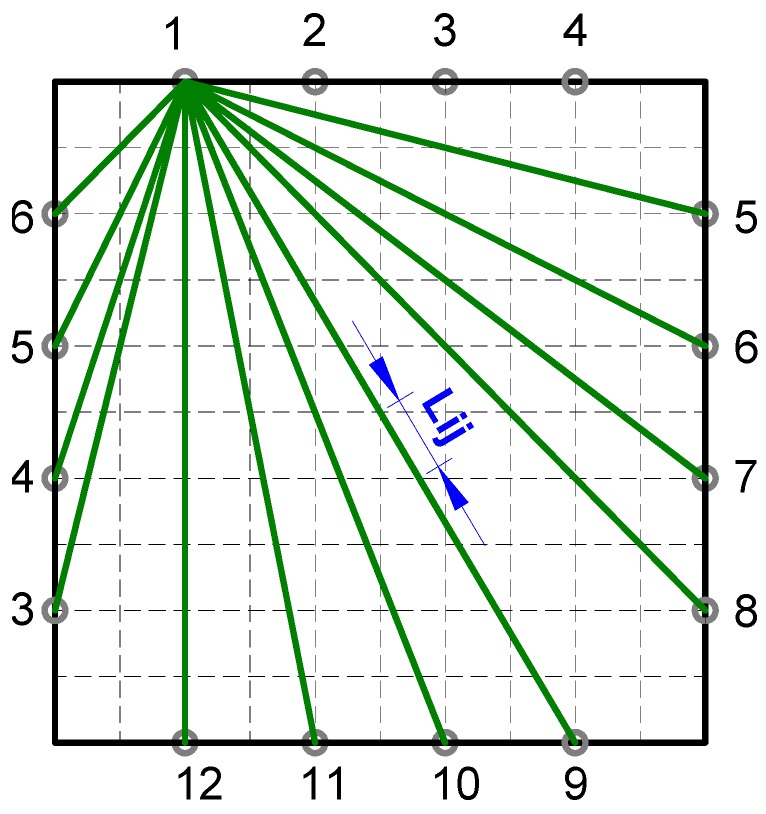
The illustration of the grids and paths in the specimen.

The plate under study was divided into grids, and the sensor recording the data at a receiver location (named projection data) can be expressed as a sum of the contributions from grids that lie on the straight path connecting the sensor to the actuator. Figure 2 illustrates a specimen divided into 100 grids with 16 PZT sensors, and 12 paths are illustrated when No. 1 PZT is regarded as the actuator. The contribution of each grid is proportional to the path length in that grid, which is regarded to be its weight. Thus, a weight matrix is constructed as a rectangular array whose size is equal to the number of paths multiplied by the number of grids. From the projections (measured data) and the weight matrix (created from sensor locations and ray geometry), the field value (correlation coefficient in this study) was obtained using the ART method as below:

The ART method was used to solve the ill-posed equation,

(7) $$A_{\left(i\times j\right)}\times x_{\left(j\times 1\right)}=b_{\left(i\times 1\right)},$$

where A represents the weight of *i*-th path in *j*-th grid, x represents the image results for each cell, and $b_{\left(i\times 1\right)}$ represents the change of signal feature (correlation coefficient is used in this study) for each path.

The $b_{\left(i\times 1\right)}$ can be expressed as

(8) $$b_{\left(i\times 1\right)}=\underset{j}{\int }X\left(i,j\right)dl_{ij},$$

where X(i,j) is the attenuation coefficient for *i*-th path in *j*-th grid, and the $L_{ij}$ represents the real length for *i*-th path projected to *j*-th grid. In this study, the X(i,j) is assumed to be proportional to the $L_{ij}$

(9) $$b_{\left(i\times 1\right)}=k\times L_{ij}x_{\left(j\times 1\right)},$$

where k is the proportional coefficient with a constant value.

(10) $$\left(\begin{array}{c} b_{1}\\ \begin{array}{c} \vdots \\ \begin{array}{c} b_{i}\\ \vdots \end{array} \end{array}\\ b_{m} \end{array}\right)=k\left(\begin{array}{ccc} L_{11} & \cdots & \begin{array}{ccc} L_{1j} & \cdots & L_{1n} \end{array}\\ \vdots & & \begin{array}{ccc} \vdots \; & & \;\vdots \end{array}\\ \begin{array}{c} L_{i1}\\ \begin{array}{c} \vdots \\ L_{m1} \end{array} \end{array} & \begin{array}{c} \cdots \\ \begin{array}{c} \cdots \end{array} \end{array} & \begin{array}{c} \begin{array}{ccc} L_{ij} & \cdots & L_{mj} \end{array}\\ \begin{array}{ccc} \begin{array}{c} \vdots \\ L_{mj} \end{array} & \begin{array}{c} \cdots \end{array} & \begin{array}{c} \vdots \\ L_{mn} \end{array} \end{array} \end{array} \end{array}\right)*\left(\begin{array}{c} x_{1}\\ \begin{array}{c} \vdots \\ \begin{array}{c} x_{j}\\ \vdots \end{array} \end{array}\\ x_{n} \end{array}\right),$$

where *m* represents the number of paths and *n* represents the number of grids.

Then, the ART method was included to solve the matrix ***x***. The classical and most known method of the ART class is called Kaczmarz’s method. The method is a so-called row action method, since each iteration consists of a “sweep” through all the rows in the matrix ***A***. The method used one equation in each step, and an iteration consists of *m* steps.

The algorithm for Kaczmarz’s method updates $x^{k}$ in the following way:

(11) $$x^{k,0}=x^{k},\;x^{k,i}=x^{k,i-1}+\lambda_{k}\frac{b_{i}-a^{i},x^{k,i-1}}{\|a^{i}{\|}_{2}^{2}},i=1,2,\ldots ,m\;x^{k+1}=x^{k,m}$$

**Figure 3:**
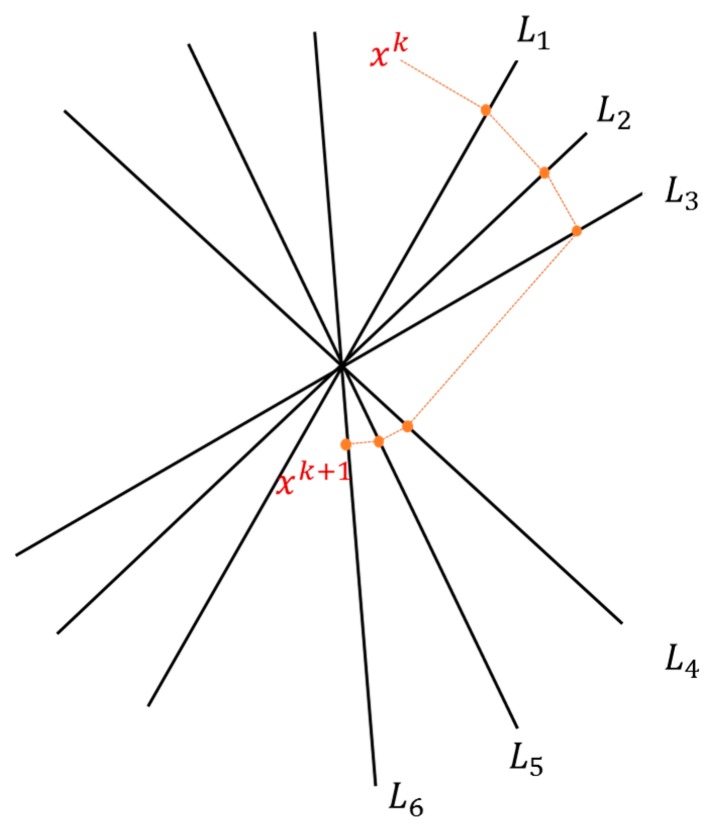
Kaczmarz’s Method.

Figure 3 shows an example of a sweep for the consistent case with the relaxation parameter$\;\lambda_{k}=1$.

## 3. Experiment Process

### 3.1. Specimen and PZT Sensor

**Figure 4:**
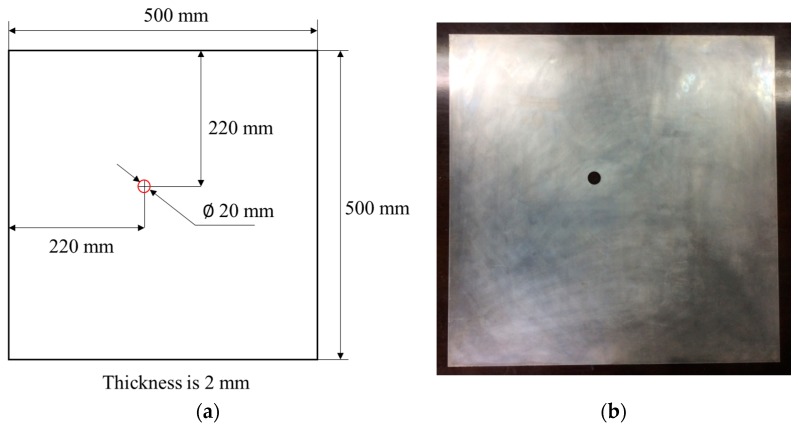
(**a**) The geometry of the specimen and (**b**) the real specimen.

**Figure 5:**
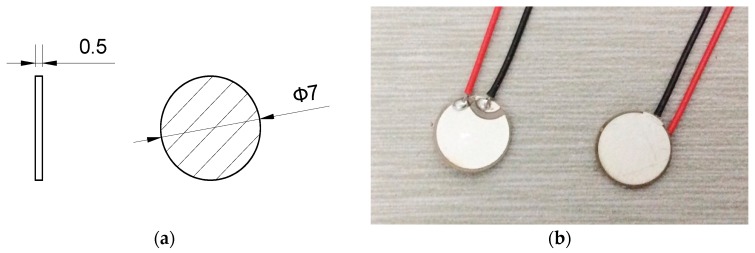
(**a**) The geometry of the PZT and (**b**) the outline of the PZT.

In the experiment, the material is Al-2024-T3; its density, Young Modulus, and Poisson’s rate are 2.78 g/cm^3^, 73.1 GPa, and 0.33, respectively. The geometry of the material is 500 mm×500 mm×2 mm, as illustrated in Figure 4. The piezoelectric sensor used in the study was manufactured by Stem, Inc. (Millbrae, CA, USA). The geometry and outline of the PZT is illustrated in Figure 5. The properties of the PZT are shown in Table 1.

### 3.2. Sensor Layout

**Figure 6:**
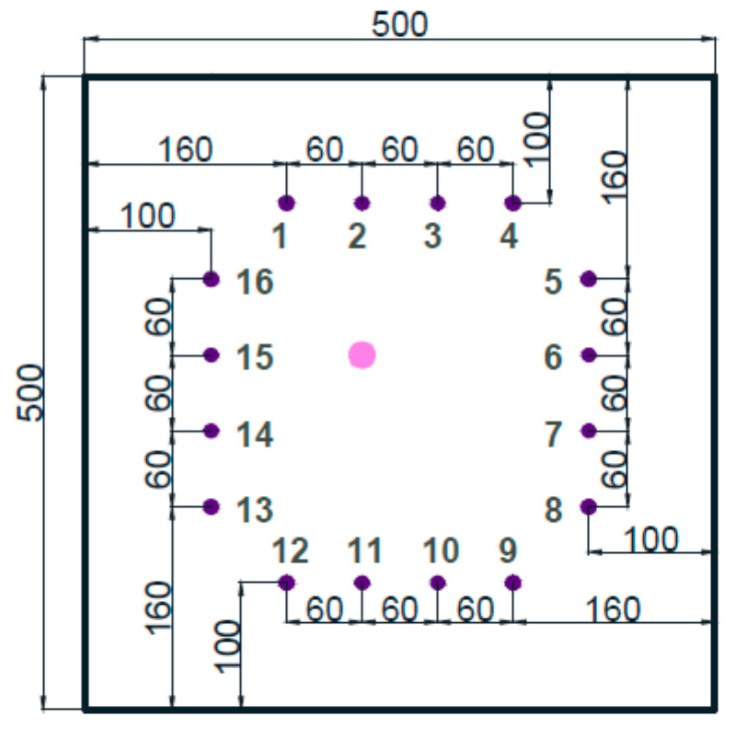
The layout of the PZT sensors. (The unit in the figure is mm.)

**Figure 7:**
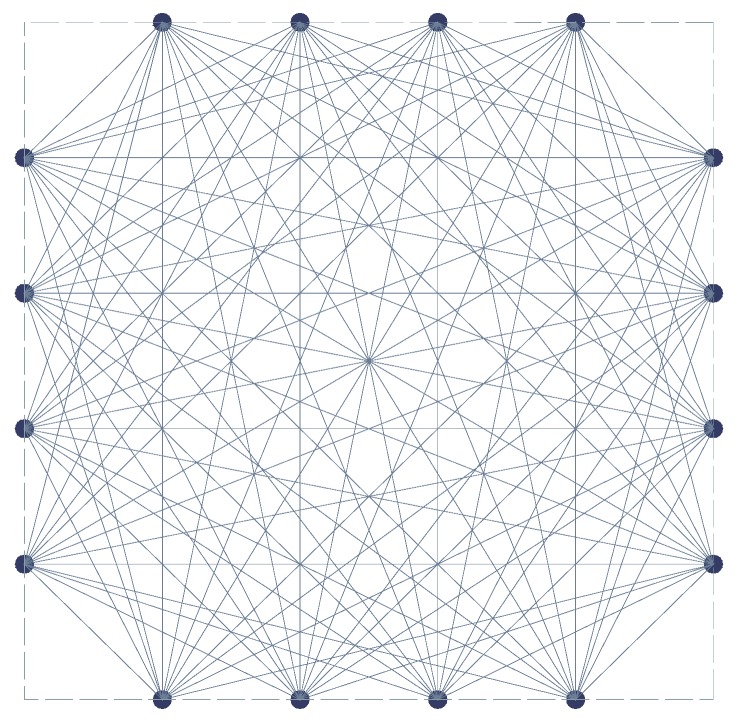
The 96 signal paths for the PZT sensors layout.

In the field of structural health monitoring, there are two configurations for PZT sensors, namely, pulse-echo configuration and pitch-catch configuration. The pitch-catch configuration was chosen in this study. In the pitch-catch configuration, the actuator and the receiver are placed across the potentially damaged region. When damage exists in the potentially damaged region, the discontinuities in the wave propagation paths will alter the characteristics of the wave. The layout of the PZT sensors is illustrated in Figure 6. This kind of layout is more efficient with limited sensors [13]. Based on this layout, 96 actuator-sensor paths for each actuator and sensor are shown in Figure 7.

### 3.3. Experimental Setup

**Figure 8:**
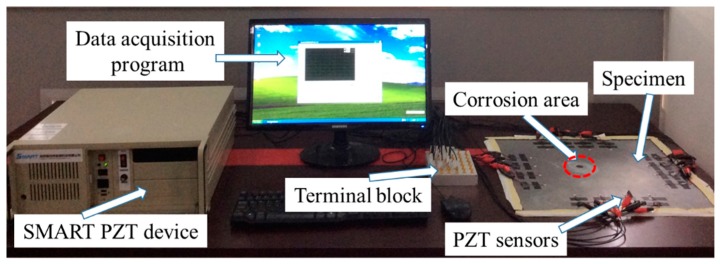
The experimental setup.

The overall experimental setup of the structural health monitoring system for hole-edge corrosion damage included the SMART PZT device, data acquisition, terminal block, PZT sensors, and specimen. A schematic of the overall procedure is illustrated in Figure 8. SMART PZT device manufactured by Nanjing SMART Company generates an excitation signal and senses the wave propagation through the specimen. The excitation signal was a five cycles tone burst generated by SMART, and the SMART is used to record both the received signals and the excitation signals.

### 3.4. Excitation Signal

The mechanism of Lamb waves damage quantification is to identify discontinuities in the wave propagation paths that alter the characteristics of transmitted/deflected waves [29], and the damage quantification results will depend on both the mode and frequency choice because of wave structure change [37]. Compared with the S_0_ mode, the fundamental antisymmetrical mode (A_0_) of Lamb waves can detect the thickness-loss damage more accurately [13,30].

**Figure 9:**
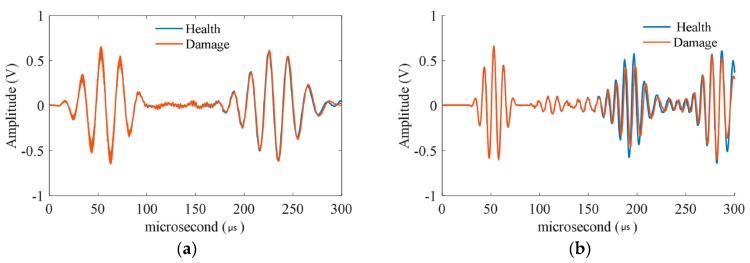

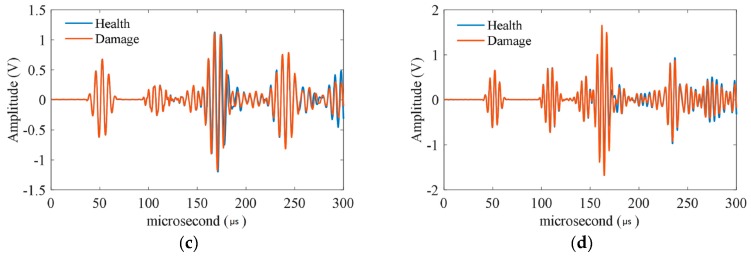
The different frequencies of the Lamb wave in a specific path based on hole-edge corrosion damage. (**a**) 50 kHz; (**b**) 100 kHz; (**c**) 150 kHz; and (**d**) 200 kHz.

Based on the sensor layout, the excitation signal should be designed. For the A_0_ mode of the Lamb wave, the phase velocity is varied by the product of frequency and thickness. In this study, the thickness of the specimen was 2 mm, and four different frequencies (50 kHz, 100 kHz, 150 kHz, and 200 kHz) of Lamb waves were compared, as shown in Figure 9, for a specific actuator-sensor path. When the frequency-thickness was 0.2 MHz·mm, the received A_0_ signal was sensitive to the varying thickness. Thus, the central frequency of the excitation signal was set to 100 kHz.

A Hamming-windowed sinusoidal tone burst with five cycles was used as the excitation signal. The expression of the excitation signal is

(12) $$u\left(t\right)=A\left[H\left(t\right)-H\left(t-\frac{N}{f_{c}}\right)\right]\times \left(1-\cos\frac{2\pi f_{c}t}{N}\right)\sin2\pi f_{c}t,\;\;\;\;\;\;\;\;\;\;\;\;\;\;\;\;\;\;\;\;\;\;\;\;\;\;\;\;\;\;$$

where $f_{c}$ is the central frequency of the signal, N is the number of crests, A is the signal amplitude, and H(t) is the Heaviside step function.

### 3.5. Imaging with Corrosion Damage

**Figure 10:**
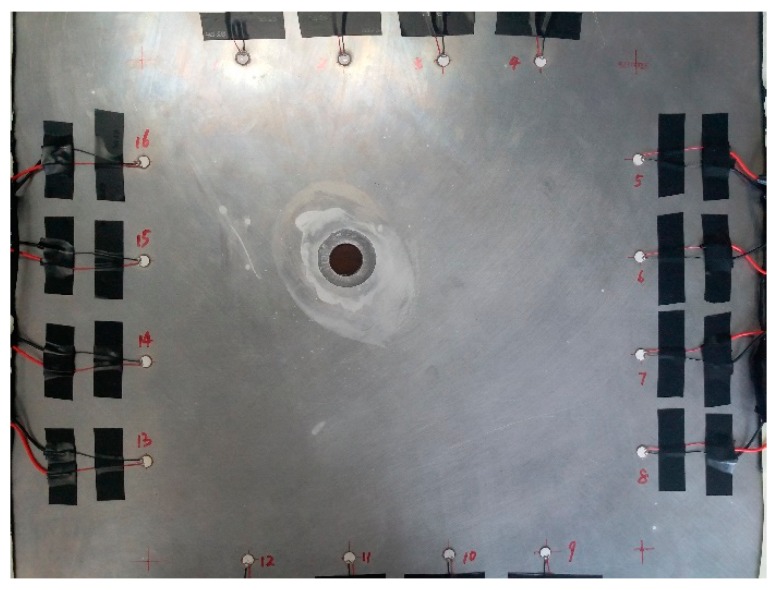
The specimen corroded by hydrofluoric acid.

Based on the setup of the experiment, the Lamb wave signals for 96 actuator-sensor paths without corrosion damage (namely healthy condition) were obtained by the SMART PZT device. Then the hole-edge damages were made artificially. In order to manufacture the artificial corrosion damage, hydrofluoric acid was used on the plate at the edge of the hole. Hydrofluoric acid (400 mL) with the concentration of 20% was used to corrode the Al-2024-T3. The detailed information of the hydrofluoric acid is illustrated in Table 2. The specimen corroded by hydrofluoric acid is shown in Figure 10.

**Figure 11:**
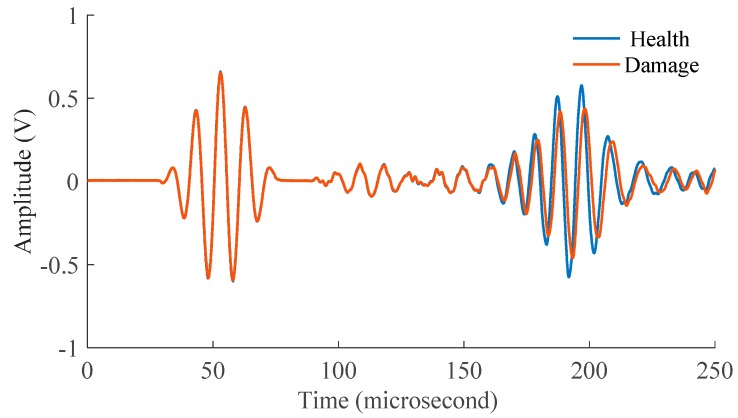
The Lamb wave signals without damage and with damage.

After corroding, the Lamb wave signals for 96 actuator-sensor paths with hole-edge corrosion damage were obtained by the SMART PZT device. In order to eliminate the measuring errors and the errors caused by the external environment, the Lamb wave signals of each actuator-sensor path were obtained three times, and the average value of signal features (the correlation coefficient was used in the study) for each set of data was used. For illustration, the signals based on the healthy and damaged conditions of one actuator-sensor path of the Lamb wave, which is across the hole-edge corrosion damage area, are shown in Figure 11. Based on the thickness-loss of aluminum, the *fd* (the product of frequency and thickness of the specimen) of the Lamb waves decreased, and the phase velocity of A_0_ mode was changed. The corrosion damage is a kind of discontinuity that can disperse and reflect energy of the original Lamb wave and cause changes in wave characteristics.

**Figure 12:**
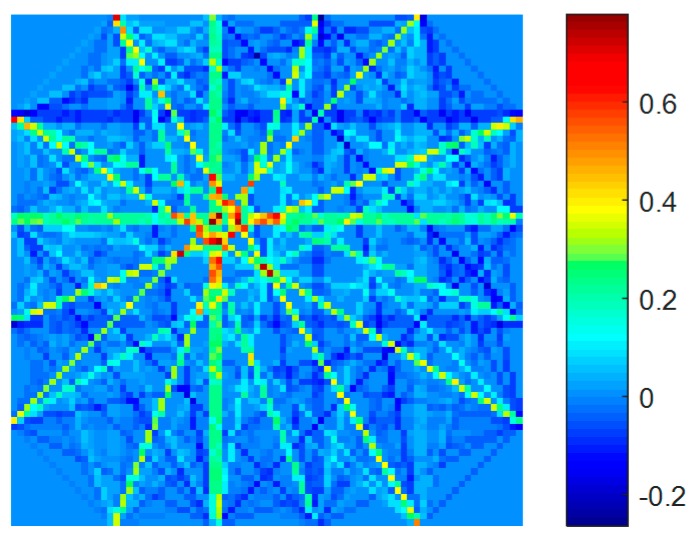
The original tomographic imaging.

**Figure 13:**
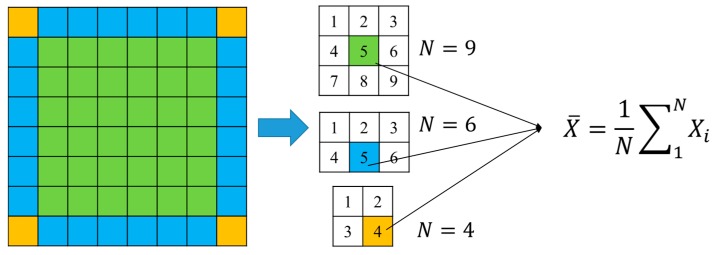
The method of homogenization to make the image smoother.

Based on the signals of the healthy condition and the signals of the damaged condition, the ART method was used to locate and quantify the hole-edge corrosion damage. In order to obtain a detailed image, 6400 square grids of the same size were divided. Based on the 96 paths, a tomographic image was obtained. The ART method was used to find the optimal solution for the ill-posed equation. Because the matrix ***A*** is sparse, the original image (as shown in Figure 12) is strengthened or weakened in the specific path. In order to make the tomographic image describe the real condition of the damage, a homogenization method was designed to make the image smoother. As illustrated in Figure 13, there are three situations: the grid in the green area, blue area, and yellow area.

(a) When the grid is in the green area, the updating step is to average the value of the surrounding eight grids with its own value.
(b) When the grid is in the blue area, the updating step is to average the value of the nearby five grids with its own value.
(c) When the grid is in the yellow area, the nearby three grids’ value are included with its own value to calculate a new value, which is regarded as updated.

**Figure 14:**
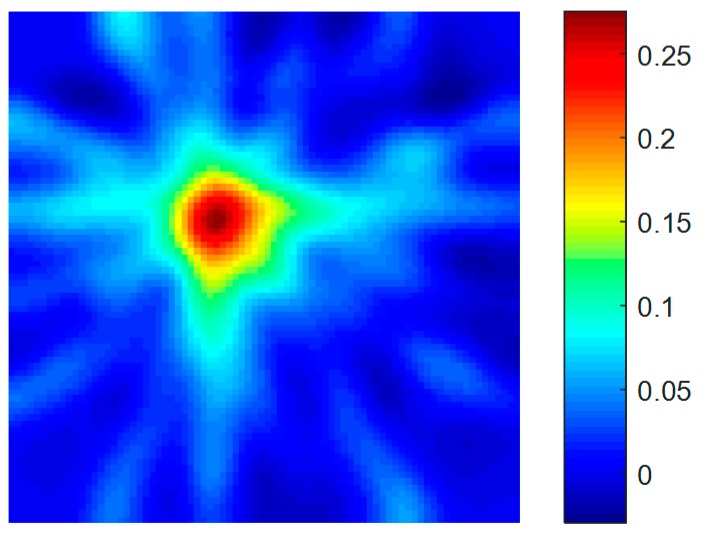
The tomographic imaging results with homogenization.

The tomographic images based on the homogenization method are illustrated in Figure 14, and it shows that the homogenization can depict the corrosion damage more accurately.

## 4. Discussion

In order to verify the proposed imaging method for monitoring the corrosion damage at the hole in the plate, the calculated results were used to compare with the real corrosion damage made by hydrofluoric acid. The relative error refers to the error between the predicted damage and the real damage. The definition of the relative error is:

(13) $$\mathrm{relative}\;\mathrm{error}=\frac{\left|D-\bar{D}\right|}{D},$$

where D is the real damage and $\bar{D}$ is the predicted damage.

**Figure 15:**
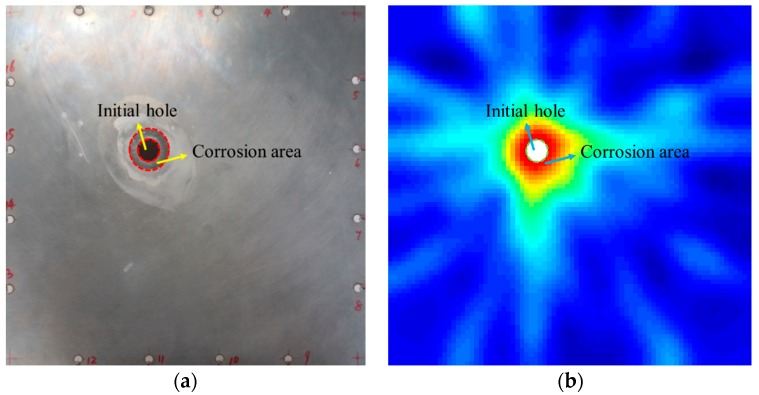
(**a**) The area of real corrosion damage and (**b**) the area of predicted corrosion damage.

In Figure 14, the red area is highlight and the threshold is set to 0.2, when the value of the grid is larger than 0.2, the grid is regarded as the corrosion damage area. After calculating, the predicted corrosion area based on the tomographic method is 768.65 mm^2^, and the real corrosion damage area in the specimen is 647.95 mm^2^. The relative error between the predicted corrosion damage and the real corrosion damage is 18.63%. The real corrosion damage and the predicted corrosion damage are illustrated in Figure 15. The relative error is less than 20% and confirms that the proposed method can locate the corrosion damage at a hole in the plate and can predict the area of the corrosion damage around the hole accurately.

The different types of damage should be distinguished accurately in practical engineering. For riveted connections in practical engineering, the rivet could be loose in the special condition, and wear between the loosening rivet and the substrate will occur. The hole size will increase based on the wear, however, the quantification of the increasing hole size can be ignored in practical engineering. For crack damage, references [38,39,40,41] show that the S_0_ mode of the Lamb wave is more sensitive to crack damage, and the damage features extracted from the Lamb wave are different from that of corrosion damage. As in reference [28], the normalized amplitude and phase change are extracted to quantify the hole-edge crack damage. In this paper, the proposed method is only used to monitor the hole-edge corrosion damage. For other types of damage, the different modes of the Lamb wave and the different damage features of the Lamb wave should be studied.

## 5. Conclusions

This study presents a technique for imaging hole-edge corrosion damage in the plate structure based on the Lamb wave tomographic method. An experimental procedure with a cross-hole layout using 16 piezoelectric (PZT) sensors was designed. The A_0_ mode of Lamb wave that is sensitive to the thickness-loss damage with a 100 kHz was selected. The iterative algebraic reconstruction technique (ART) method was included to locate and quantify the hole-edge corrosion damage. Hydrofluoric acid with the concentration of 20% was used to corrode the specimen artificially. To estimate the effectiveness of the proposed method, the real corrosion damage was used to compare with the results of the tomographic method. Results show that the Lamb wave-based tomographic method can (1) indicate whether corrosion damage at the edge of the hole exists in the structure; (2) locate the hole-edge corrosion damage accurately; and (3) quantify the area of the hole-edge corrosion damage, and the relative error is less than 20%.

In the future, the monitoring of the corrosion growth for a variety of growth levels from tiny to large will be studied, and the monitoring experiment will be developed soon. In order to study the different conditions of hole-edge corrosion damage, multi-hole and different hole locations in plate structures will be included. The corrosion condition in practical engineering will also be studied in the next step.

## Figures and Tables

**Table 1:** The properties of PZT sensor.

**Product ID**	SMD07T05R412WL
**Material**	SM412
**Geometry**	Diameter: 7 mm Thickness: 0.5 mm
**Resonant Frequency $f_{r}$**	4.25 MHz ± 5%
**Electrostatic Capacity $C_{S}$**	2.5 nF ± 30%
**Test Condition**	25 °C ± 3 °C

**Table 2:** The detailed information of hydrofluoric acid.

**Molecular Formula**	**HF**	
**Content of HF**	≥40%	
**Impurity Content (%)**	Fe	≤0.0001
	Cl	≤0.001
	PO_4_	≤0.0002
	Heavy metal (Pb)	≤0.0005
	Fluorosilicate (SiF_6_)	≤0.04
	Others	≤0.004
